# An Examination by Site-Directed Mutagenesis of Putative Key Residues in the Determination of Coenzyme Specificity in Clostridial NAD^+^-Dependent Glutamate Dehydrogenase

**DOI:** 10.4061/2011/595793

**Published:** 2011-08-16

**Authors:** Joanna Griffin, Paul C. Engel

**Affiliations:** School of Biomolecular and Biomedical Science, Conway Institute, University College Dublin, Belfield, Dublin 4, Ireland

## Abstract

Sequence and structure comparisons of various glutamate dehydrogenases (GDH) and other nicotinamide nucleotide-dependent dehydrogenases have potentially implicated certain residues in coenzyme binding and discrimination. We have mutated key residues in *Clostridium symbiosum* NAD^+^-specific GDH to investigate their contribution to specificity and to enhance acceptance of NADPH. Comparisons with *E. coli* NADPH-dependent GDH prompted design of mutants F238S, P262S, and F238S/P262S, which were purified and assessed at pH 6.0, 7.0, and 8.0. They showed markedly increased catalytic efficiency with NADPH, especially at pH 8.0 (*∼*170-fold for P262S and F238S/P262S with relatively small changes for NADH). A positive charge introduced through the D263K mutation also greatly increased catalytic efficiency with NADPH (over 100-fold at pH 8) and slightly decreased activity with NADH. At position 242, “P6” of the “core fingerprint,” where NAD^+^- and NADP^+^-dependent enzymes normally have Gly or Ala, respectively, clostridial GDH already has Ala. Replacement with Gly produced negligible shift in coenzyme specificity.

## 1. Introduction

The alternative nicotinamide cofactors, NAD(H) and NADP(H), involved in many reactions catalysed by dehydrogenases and reductases, differ only in the presence or absence of a phosphate group esterified to the 2′-hydroxyl group of the AMP moiety, and the two oxidised coenzymes undergo the same 2-electron transfer to give the 1,4-dihydropyridine form. There is, however, a general metabolic separation of the roles of these chemically similar cofactors, and accordingly most dehydrogenases are specific for one or other coenzyme pair. Glutamate dehydrogenase (GDH) offers examples of both specificities, with NAD^+^-dependent members of the family (EC 1.4.1.2) responsible for the catabolic oxidative deamination of L-glutamate, and NADP(H)-dependent GDHs (EC 1.4.1.4) involved in biosynthetic reductive amination, incorporating ammonia into organic combination [1, 2]. There are, however, also “dual specificity” (presumably amphibolic) GDHs (EC 1.4.1.3), especially in higher animals and in Archaea, and the GDH group thus provides a particularly interesting context within which to consider both the structural basis and the evolution of coenzyme specificity, with a complete spectrum in terms of the stringency of the preference. 

The availability of solved structures for binary complexes of other dehydrogenases with nicotinamide cofactors and the recognition of an underlying similarity between the nucleotide binding domains of different dehydrogenases led to interpretations of the basis of coenzyme binding and discrimination [3–5] and a broad consensus regarding the main determinants of specificity. Thus, coenzyme specificity for many dehydrogenases is accepted to result from specific features of a common *βαβ* fold in the dinucleotide-binding domain first described by Rossmann et al. [6]. This fold is associated with an amino acid sequence “fingerprint” [7]. Wierenga et al. [8] recognised essential structural functions of several side-chains of this *βαβ*-fold in five NAD^+^-dependent dehydrogenases and highlighted the conserved sequence Gly-X-Gly-X-X-Gly. This “core fingerprint” for coenzyme binding consists of residues that form a hydrophobic core between the *β*-strands and the *α*-helix, and glycine residues that allow for a tight turn between the first *β*-strand and the following *α*-helix. In all these enzymes, the last residue in helix *α*A has to be a Gly, the first Gly of the consensus, because any larger residue would interfere with the adenine ribose. The *α*-helix is believed to provide a dipole, with the fractional positive charge on its N-terminus helping to anchor the pyrophosphate moiety, and the middle one of the three conserved glycines allows for a close approach of these two partners. Further along the sequence, an aspartate, usually the last residue of *αβ*, forms hydrogen bonds with the 2′-OH and 3′-OH of the adenine ribose and thus is a significant contributor to NAD^+^ binding and recognition. The residues of the “core fingerprint” are often labelled P1 to P6, with P7 being the conserved acidic residue [9]. In all, the sequence from P1 to P7 includes 11 amino acid residues.

In NADP^+^-binding enzymes, by contrast, the third Gly of the consensus is often replaced by alanine [5, 7, 8]. Also, electrostatic repulsion by the acidic sidechain at P7 is regarded as one of the principal means by which NAD^+^-specific enzymes discriminate against NADP^+^, and so NADP^+^-dependent dehydrogenases usually have a smaller, uncharged side chain at P7 to admit the negatively charged phosphate group. Instead, a positively charged side-chain is found nearby for binding the phosphate.

These generalised observations have prompted numerous attempts, with varying degrees of success, to switch the coenzyme specificity of dehydrogenases (e.g., [5, 11–16]). Some groups, attempting to switch from NAD^+^ to NADP^+^ specificity, have focussed on altering the P7 acidic residue to a smaller uncharged amino acid and/or introducing positive charge in order to stabilise the 2′-phosphate. Nishiyama et al. [13], working with NAD^+^-dependent malate dehydrogenase from *T. flavus*, were guided by crystallographic information and alignment with NADP^+^-dependent homologues, and made more extensive mutational changes. Their reasonably successful results emphasise the importance of creating space for the phosphate group of the larger coenzyme. 

Sometimes changed coenzyme activity ratios following mutagenesis merely reflect decreased affinity for the original coenzyme rather than increased activity with the target coenzyme (e.g., Huang et al. [14]). On the other hand, a study of NADP^+^-dependent glutathione reductase and NAD^+^-dependent lipoamide dehydrogenase [5, 11] represented a notable success. In *E. coli* glutathione reductase, P7 is neutral valine, and positive residues nearby provide a cushion for the negatively charged 2′-phosphate group of NADP^+^ [17–19]. The pyrophosphate bridge of the coenzyme is bound at the C-terminal end of a parallel *β*-sheet in the NADP(H) domain, its negative charge stabilized by the dipole at the N-terminal end of an *α*-helix. Conversely, NAD^+^-specific lipoamide dehydrogenase, with a homologous structure [20, 21] and similar chemistry, shows all the characteristic structural features noted above. By systematically addressing these features, Perham and colleagues reversed the coenzyme preference in both cases. More recently Rodríguez-Arnedo et al. [22] successfully reversed the coenzyme specificity of isocitrate dehydrogenase from *Haloferax volcanii* using sequence alignment of several isocitrate dehydrogenases without the help of a 3D-structure. A protein containing 5 mutations showed a complete switch of specificity from NADP^+^ to NAD^+^. Ehsani et al. [23] also achieved reversal of the coenzyme specificity of 2,3-butanediol dehydrogenase from *Saccharomyces cerevisiae*. Using in this case both sequence studies and the crystal structure of the homologous NADP(H)-dependent yeast alcohol dehydrogenase.

The glutamate dehydrogenase family, however, displays unusual characteristics, calling into question some of the previous generalisations, and indeed it has been suggested that this family may offer more than one structural solution to the challenge of achieving a particular specificity [24]. At P7, for instance, the NAD^+^-dependent GDHs from *Peptostreptococcus *(now reclassified as *Peptoniphilus*)* asaccharolyticus* [25] and *Clostridium difficile* [26] have the predicted acidic sidechain (glutamate residue). However, P7 is also glutamate in dual-specific GDHs [1], and, even more remarkable, the NADPH-dependent GDHs, such as that from *E. coli *[27, 28], have aspartate at this position. Moreover, GDH from *Clostridium symbiosum*, also strongly NAD^+^-specific, has glycine (G261) at P7 [29] instead of a negative residue. Contemplating this variability, Baker et al. [30] proposed that an acidic residue at P7 in the NADPH-dependent GDHs might shift the pK of the 2′-phosphate, so that a protonated phosphate oxygen atom could act as a hydrogen-bond donor to the carboxyl side-chain at P7. They postulated two modes of NADPH binding, distinguished by the preference for either monobasic or dibasic 2′-phosphate. The type of residue at P7 would indicate the class into which any particular enzyme falls. It is possible that a glycine residue can occupy this position in the clostridial enzyme, since the 2′-hydroxyl of the adenine ribose is hydrogen-bonded instead to the side-chain of N290 [9]. 

In the light of all these speculations, we have used site-directed mutagenesis to test the importance of postulated key residues in determining coenzyme specificity of the NAD^+^-dependent GDH of *Clostridium symbiosum* (CsGDH), for which there is a cloned gene [29] and high-resolution crystal structures [9, 31], including that of the enzyme-NAD^+^ complex. 

**Figure 1:**
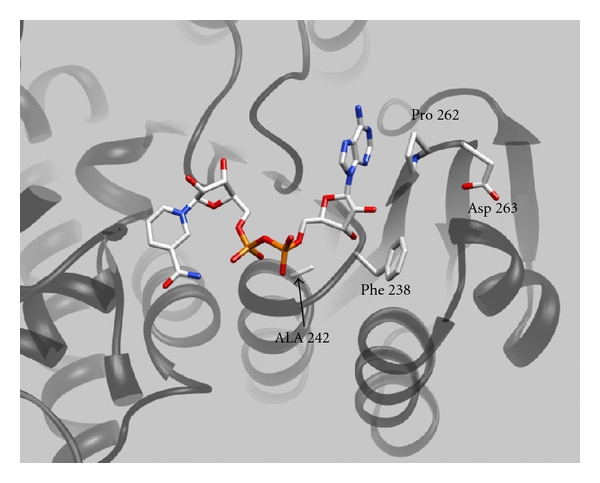
Coenzyme binding to clostridial glutamate dehydrogenase. The figure shows NAD^+^ bound to the coenzyme binding domain of glutamate dehydrogenase of *Clostridium symbiosum*. The image, made using PYMOL, is from the coordinates of the solved structure of the Enzyme-NAD^+^ complex [9] and highlights the various contact residues mutated in this study.

Sequence alignment of clostridial GDH with NADPH-dependent GDHs showed that most NADPH-dependent GDHs have serine residues at the positions corresponding to F238 and P262 in the clostridial enzyme. Since these residues are found in the coenzyme binding pocket (Figure 1), it was postulated that the rings of F238 and P262 may limit the size of the pocket that might otherwise accommodate the extra phosphate on NADPH [9, 30]. F238 and P262 were therefore replaced with serine residues to create more space and provide stabilizing hydrogen bonds for the 2′-phosphate group. 

**Figure 2:**
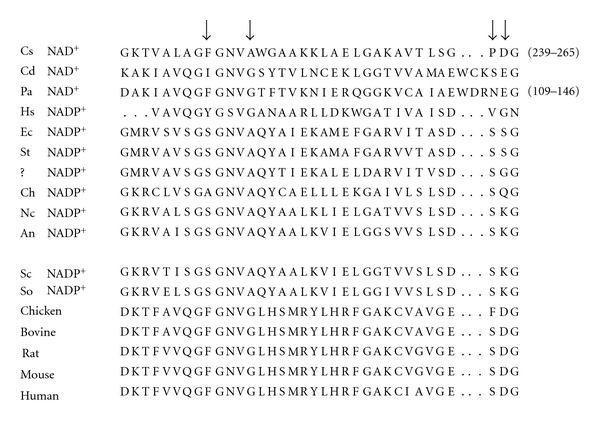
Alignment of the relevant stretch of amino acid sequence in a number of different GDHs. The arrows indicate the three positions targeted for mutagenesis in this study and the numbers in brackets the residue numbers. The abbreviated biological species are Cs *Clostridium symbiosum*, Cd *Clostridium difficile*, Pa *Peptostreptococcus asaccharolyticus*, Hs *Halobacterium salinarum*, Ec *Escherichia coli*, St *Salmonella typhimurium, ? *unidentified bacterium, Ch *Chlorella sorokiniana*, Nc *Neurospora crassa*, An *Aspergillus nidulans*, Sc *Saccharomyces cerevisiae*, So *Schwanniomyces occidentalis*. The coenzyme specificity of the source GDH is indicated for all the monospecific enzymes, the others being dual-specific.

The first residue on the *β*-sheet following the *βαβ* fold in (CsGDH) is D263 (Figure 1). At this position, the NADPH-dependent GDHs either have serine as in *E. coli*   [27] and *Salmonella typhimurium* [32], or else positively charged lysine as in *Neurospora crassa *[33–35], *Aspergillus nidulans* [36], *Saccharomyces cerevisiae* [37], and *Schwanniomyces occidentalis* [38] (Figure 2). Lysine is also found in the dual-specific enzyme from *Pyrococcus furiosus* [39]. D263 of clostridial GDH was, accordingly, replaced with a lysine.

Finally, the P6 alanine at position 242 was replaced by the glycine more usually expected in NAD^+^-dependent enzymes. 

## 2. Experimental

### 2.1. Mutagenesis

Four single mutants, F238S, P262S, D263K, and A242G were constructed by site-directed mutagenesis using the “Quikchange” kit from Stratagene Inc. The Phe codon TTT was replaced by TCT (Ser), the Pro codon CCA with TCA (Ser), the Asp codon GAC with AAG (Lys), and the Ala codon GCA by GGA (Gly). Double-stranded plasmid DNA was extracted from *E. coli *TG1 cells harbouring a ptac85 vector containing the wildtype *Clostridium symbiosum gdh *gene [29]. This was used as the template for the mutagenic PCR sequence, 18 cycles, each comprising 30-second denaturation at 95°C, 1-minute annealing at 55°C, and 12-minute polymerisation at 68°C with high-fidelity Pfu polymerase. The mutagenic oligonucleotides, synthesised commercially by Genosys (UK), are listed in Table 1 (in the case of the A242 position, the primers were designed also to encode the A242V substitution; although this was successfully carried out, the results were not felt to be of sufficient interest to include in this paper). The double mutant F238S/P262S was constructed by extracting the DNA encoding the F238S enzyme and mutating it further with the primer for the P262S mutation. 

In all cases, original unmutated template DNA was digested with *DpnI *(1 h at 37°C) and the mutated genes in the ptac-85 vector were transformed into *E. coli* XL1-Blue cells. Selected colonies were grown overnight and plasmids were purified using Wizard minipreps. The presence of the (mutated) *gdh* gene was checked by running the DNA on minielectrophoresis gels with and without digestion by *Bam*H1 and* Sal*1. The mutations and absence of any additional changes were confirmed by sequencing of the DNA by Cytomix Ltd. 

### 2.2. Expression and Purification of Mutant Enzymes

In each case, a 1 mL overnight culture of transformants of *E. coli* XL1-Blue containing the mutated gene was inoculated into 2 × 500 mL LB broth in 2 L flasks, supplemented with 10 *μ*g/mL ampicillin and 0.5 mM IPTG. Bacterial cells, grown at 28°C for A242G, P262S, and the double mutant and 37°C for F238S, were harvested by centrifugation and disrupted by sonication. After recentrifugation, supernatants and pellets were run on SDS-PAGE gels to check that the overexpressed protein was soluble. In an alternative procedure to avoid formation of inclusion bodies in the cases of the double mutant and D263K mutant, 10 mL overnight culture was diluted 50X into TB phosphate medium supplemented with 100 *μ*g/mL ampicillin, grown at 37°C to A_500_ of ~1, and then cooled to approximately 8°C; after 30 min, 0.1 mM IPTG was added and the cells were incubated overnight (approximately 16 hours), still at 8°C. Harvested cells were processed as above. The mutant enzymes and the recombinant wildtype clostridial GDH were all purified by dye-ligand chromatography on Remazol Red Sepharose [40].

### 2.3. Examination of Coenzyme Specificity

Kinetic parameters *k*_cat_ and apparent *K*_*m*_ were obtained by measuring initial rates of reaction for the mutant and wildtype enzymes in 0.1 M potassium phosphate (pH 6.0, 7.0, and 8.0). NAD(P)H concentrations were varied over the range 0.001–0.3 mM with fixed concentrations of 20 mM 2-oxoglutarate and 100 mM ammonium chloride. Activity at 25°C was measured either by recording the decrease in A_340_ with a Kontron Uvikon 941 or Cary 50 recording spectrophotometer or, for the lower coenzyme concentrations, fluorometrically (Hitachi F-4500) with excitation and emission at 340 nm, and 450 nm, respectively.

## 3. Results

### 3.1. Overexpression and Purity of the Mutant Enzymes

**Figure 3:**
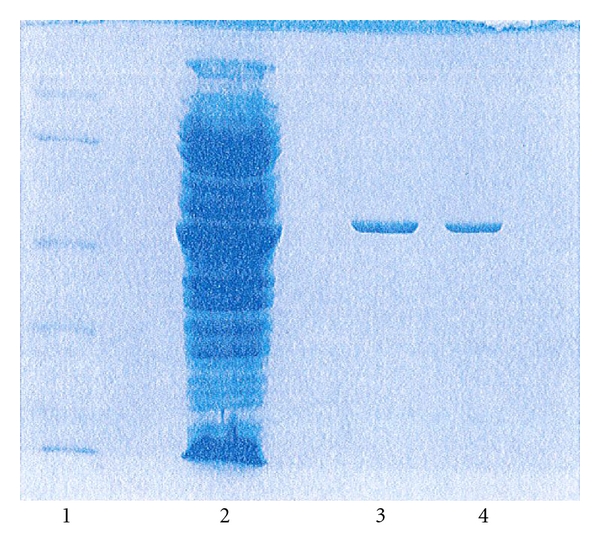
10% SDS PAGE illustrating overexpression and purification of D263K enzyme. Lane 1, molecular weight markers. Lane 2, overexpressed D263K. Lanes 3 and 4, purified D263K.

The mutated genes were in the ptac-85 vector [30], which has an IPTG-inducible *tac* promoter to overproduce the mutant protein. 10% SDS-PAGE of the soluble and insoluble samples showed that, for F238S and P262S and A242G single mutants, the overexpressed protein was soluble at 37°C. However, in the case of the double mutant and the D263K mutant, overexpressed protein was located in the pellet in the form of insoluble inclusion bodies. Lowering the growth temperature to 28°C served to solubilise the F238S/P262S protein whilst the cold overexpression method at 8°C resulted in abundant production of soluble D263K mutant protein. After purification, each mutant protein exhibited a single prominent band on SDS-PAGE with equivalent subunit molecular mass to wildtype GDH. From 1 L overnight-grown culture, 80–100 mg pure enzyme was obtained for the wildtype, approximately 50 mg for the single mutants and 40 mg for the double mutant enzyme.  Figure 3 is a representative SDS-PAGE gel showing for the D263K mutant the level of overexpression in the crude cell extract (Lane 2) and the final state of purity (Lanes 3 and 4).

### 3.2. Kinetics

**Figure 4:**
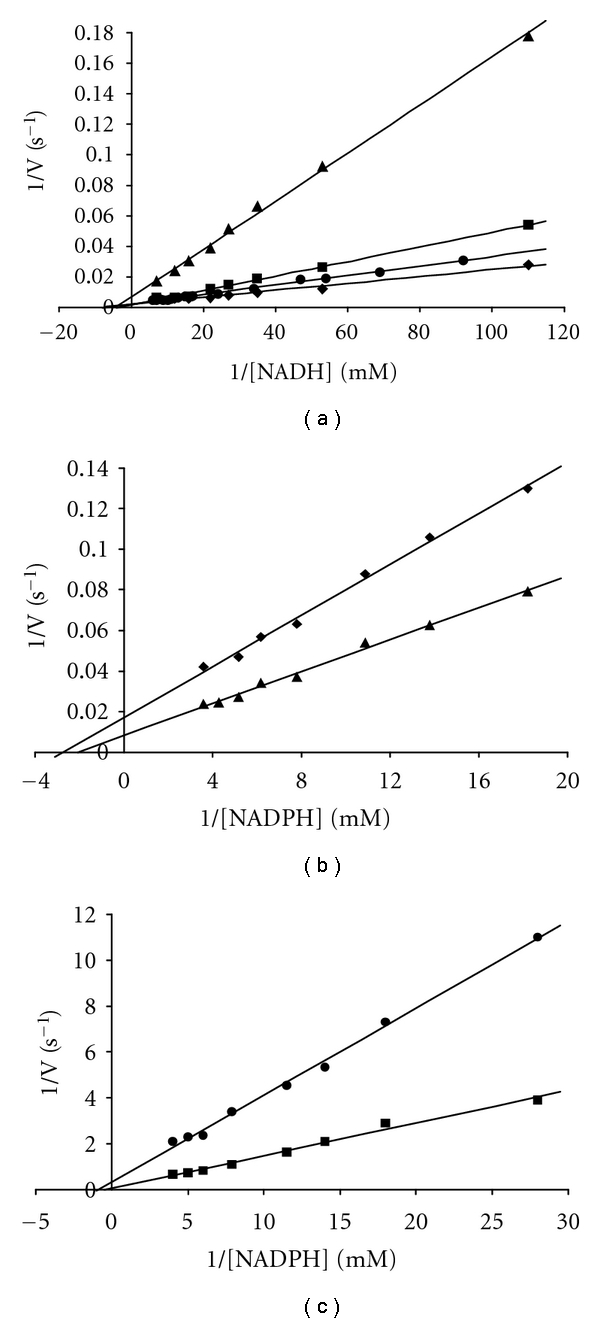
Lineweaver-Burk plots of initial rates for wildtype Cs GDH • and mutants F238S ■, P262S ♦, and F238S/P262S ▲ as a function of varying NADH (a) and NADPH (b and c) concentrations at pH 8.0.

In all cases straightforward Michaelis-Menten behaviour was observed, in keeping with the previous report [40] that the 3-substrate reaction with this enzyme is not complicated by regulatory behaviour.  Figure 4 shows a set of typical plots, in this case for pH 8 and for both coenzymes, NADH and NADPH, with the wildtype enzyme, the single mutants F238S and P262S and the double mutant F238S/P262S. Values of *k*_cat_, *K*_*m*_, and *k*_cat_/*K*_*m*_ for the reduced coenzymes at pH 6, 7, and 8 are presented in Table 2. In all cases these are “apparent” constants, not extrapolated to saturating concentrations of the other substrates, although the fixed substrate concentrations were relatively high. With the unmutated wildtype enzyme and NADH as coenzyme there was a 12-fold increase in *k*_cat_ between pH 6 and pH 8. The *K*_*m*_ for NADH was lower at pH 7 (20 *μ*M) than at either pH 6 (36 *μ*M) or pH 8 (78 *μ*M). With NADPH, however, there was a markedly different pattern, with *k*_cat_ decreasing 4.4-fold from pH 6 to pH 8 and *K*_*m*_ increasing 9-fold over the same range. As a result of these combined trends, the discrimination between the nonphosphorylated and phosphorylated cofactors is highly pH dependent. This factor, calculated as the ratio between values of *k*_cat_/*K*_*m*_ for NADH and NADPH, is 1930 (3000/1.5) at pH 8, 203 (6500/32) at pH 7, and only 9.4 (550/59) at pH 6. 

A242G showed a decreased overall catalytic efficiency for NADH at all pH values studied as compared to the wildtype enzyme. It did, however, have comparable *K*_*m*_ values. This mutation had a severe effect on the overall catalytic efficiency with NADPH as coenzyme. At pH 6 there was a 40% reduction in *k*_cat_ while *K*_*m*_ values were comparable. At pH 7 there was a 15-fold increase in *K*_*m*_ and at pH 8 rates were too low to measure, presumably owing to a further increase in *K*_*m*_ (Table 2)_._

F238S showed an unexpected increase (roughly 2-fold) in *k*_cat_ for NADH at pH 8. Nevertheless, the overall catalytic efficiency of F238S with NADH was lower at all three pH values owing to an increased *K*_*m*_ at pH 7 and 8, and the decreased value for *k*_cat_ at pH 6. In the case of NADPH, *k*_cat_ also increased (only 1.4-fold at pH 6, but about 4-fold at pH 7 and 8; Table 2). The improvements in *k*_cat_ at pH 7 and 8 were also accompanied by opposing increases in *K*_*m*_, but the overall catalytic efficiency for this mutant with NADPH was nevertheless higher than that of the wildtype enzyme at pH 7 (1.5-fold) and 8 (5-fold) resulting in a modest overall change in specificity in the direction of NADPH (Table 2).

Much more substantial changes in specificity were seen with the P262S mutant. With NADH the changes broadly follow the pattern of the F238S mutant, with increases in *k*_cat_ of 2.1-fold at pH 8 roughly counterbalanced by increases in *K*_*m*_ (Table 2). However, this mutation produced much larger changes with NADPH. At all three pH values tested, *k*_cat_ values were greatly increased, 4-fold at pH 6, 22-fold at pH 7, and 108-fold at pH 8. At pH 6 the overall catalytic efficiency nevertheless actually decreased compared to the wildtype enzyme owing to an 18-fold increase in *K*_*m*_. At pH 7, however, the *K*_*m*_ was comparable to the wildtype value, and at pH 8 it was approximately 40% lower. As a result, the overall catalytic efficiency of this mutant with NADPH was 17-fold higher at pH 7 and 172-fold higher at pH 8, as compared with the unmutated enzyme. 

With the F238S/P262S double mutant, (Table 2) the *k*_cat_ values for NADH were decreased only 1.2-fold at pH 7 and 1.7-fold at pH 8. Again, however, an increase in *K*_*m*_ resulted in decreased overall catalytic efficiency. This mutant also had a high catalytic efficiency with NADPH as coenzyme (Table 3). The double mutant showed a greater increase in *k*_cat_ over wildtype than either of the single mutants, 29-fold at pH 7 and 173-fold at pH 8. Although these increased *k*_cat_ values were accompanied by further moderate increases in *K*_*m*_, the combined effect of these changes was a 640-fold switch in coenzyme specificity at pH 8 from an NADH : NADPH ratio of 1930 : 1 for wildtype to 3 : 1 for the double mutant. A > 4-fold decrease in efficiency for NADH was accompanied by a 144-fold increase in efficiency for NADPH, in keeping with the original intention of the mutagenesis experiments.

The D263K mutation produced remarkably little change in the kinetic parameters for NADH at the three pH values examined. However, with NADPH an entirely different pattern was seen: at all three pH values the *k*_cat_ for the mutant was much higher than for wildtype GDH, and this factor increased from 5.6 at pH 6 to 9.2 at pH 7 and 46 at pH 8. As mentioned above, in the wildtype enzyme there is a strong pH dependence in the level of discrimination between NADH and NADPH. One of the outcomes of the D263K mutation was to largely remove this pH sensitivity over the range pH 6 (2.3-fold discrimination) to 8 (7.1-fold). 

## 4. Discussion

In examining the kinetic results for the wildtype clostridial enzyme, it is interesting to note the opposite pH dependence for the two cofactors. With NADPH, increasing pH from 6 to 8 raises the *K*_*m*_ and decreases *k*_cat_. This is in line with prediction, since over this pH range the phosphate should undergo deprotonation, increasing its local negative charge and thus making it more difficult to bind at the intended site and presumably also to achieve a catalytically productive orientation when bound. Accordingly also, the discrimination between the two cofactors is much more pronounced at pH 8 (nearly 2000-fold) than at pH 6 (9.4-fold). Kinetic parameters show that all three single mutants and the double mutant had increased overall catalytic efficiency with NADPH as predicted. However, the improvements, where seen, were in *k*_cat_ values, and not in *K*_*m*_ values as might have been anticipated. One must therefore consider the possibility that the wildtype structure allows after all a binding of NADPH that is reasonably tight but is catalytically unfavourable or inactive. The mutations then may not tighten overall binding but nevertheless might increase the fraction of bound molecules that are in a catalytically productive orientation. 

This suggestion is strongly supported by measurements of coenzyme dissociation constants. These measurements, made by studying each coenzyme's ability to protect the active site –SH of Cys-320 against modification by DTNB at pH 7 [41], provided startling results. First of all, the dissociation constants of the wildtype enzyme for NADH and NADPH were virtually identical, 23 *μ*M and 24 *μ*M, respectively, suggesting that low activity with NADPH reflects not so much weak binding as unproductive binding. Secondly, the mutations resulted in weaker binding of both coenzymes, in some cases markedly so. Thus, for example, P262S, which shows the best kinetic improvement with NADPH at pH 7, shows a 2-fold increase in *K*_*d*_ for NADH (52 *μ*M) and 16-fold increase for NADPH (375 *μ*M). Thus, in this case, but also in the others, in purely binding terms, the mutations appear to discriminate even further against NADPH. This finding contains an important lesson for protein engineering, namely, that a substrate molecule may have more than one binding mode, and, in seeking to optimise performance, it is desirable to weaken unproductive binding as well as to strengthen the catalytic binding mode. One obvious caveat is that the binding measurements are for formation of binary complexes, and it is entirely possible that the presence of glutamate could alter the distribution between different coenzyme binding modes. It would nevertheless now be very interesting to see crystal structures for both NADH and NADPH bound to the wildtype enzyme and to one or more of these mutants. It is also important, in considering what appear at first sight to be obvious contradictions between kinetic results and measurements of dissociation constants, to recognise that we have as yet no proper analysis of overall kinetic mechanism for this GDH. Correspondingly we have no basis for assuming the relationship between the *K*_*m*_ value for a coenzyme and the *K*_*d*_ for its binding to form a binary complex. If, for example, like bovine GDH [42, 43], this enzyme follows a rapid-equilibrium random-order pathway, then the *K*_*m*_ should be equated instead with the *K*_*d*_ for dissociation from the ternary enzyme-glutamate-coenzyme complex. It is reasonable to expect some synergy in binding of the two substrates, and this could then account for cases where the measured *K*_*d*_ is substantially higher than the *K*_*m*_ value determined here.

It is also noteworthy that all the single mutants and the double mutant can still function well with NADH as coenzyme, with the overall catalytic efficiency of P262S being higher than that measured for the wildtype at pH 6 and 8. These increases in catalytic efficiency were largely due to increases in *k*_cat_, as *K*_*m*_ values for all mutants were increased using NADH as coenzyme at all pH values measured. The intended shift in discrimination between the two coenzymes has thus been achieved not by disfavouring the activity with NADH, but almost entirely by increasing the activity with NADPH. 

The beneficial effects of the D263K mutation in terms of improved acceptance of NADPH relative to NADH are almost entirely due to substantial shifts in *k*_cat_ values, and once again there is no simple correlation between changes in values of *K*_*d*_ for the binary enzyme-coenzyme complexes and the corresponding effects on *K*_*m*_ values. Whilst there is little change in the *K*_*m*_ values at pH 7, the *K*_*d*_ values for both coenzymes are increased (5-fold for NADH and 10-fold for NADPH). Aspartic acid at position 263 in the clostridial enzyme is equivalent to a serine in the *E. coli* enzyme and a glutamate in the *Peptostreptococcus* (*Peptoniphilus*) *asaccharolyticus* NAD^+^-dependent GDH. The importance of this position has also been examined in the latter enzyme by site-directed mutagenesis [42]. Replacement of E145 by lysine again produced a shift in specificity in the direction of NADPH, but less pronounced than for the clostridial enzyme and certainly not resulting in a good catalyst. In the case of the *P. asaccharolyticus* GDH, replacing the P7 Glu by Lys resulted, much more impressively, in a genuinely dual-specificity GDH about 20-fold less active than the wildtype with NADH. Lysine, however, is not found at this position in any of the GDH sequences studied to date, regardless of coenzyme specificity, and as already noted, in clostridial GDH this position is occupied not by a negative residue but by glycine. 

If the “fingerprint rules” were to be applied, the A242G mutant might perhaps have been expected to use NADH at an even greater rate than the wildtype enzyme. This clearly was not the case: replacement of A242 with glycine resulted in a mutant showing considerably decreased catalytic efficiency with both NADH and NADPH. These results do, however, confirm the importance of the P6 position in coenzyme binding.

Although this programme of mutagenesis has been guided by a high-resolution structure, it is important to bear in mind (a) that a binary complex structure is available only for the enzyme-NAD^+^ complex [9] and (b) that no structure is available for a ternary complex of clostridial GDH. It is known, however, that the binding of glutamate produces a considerable hinge movement, tending to close up the active-site cleft [31]. It seems very likely that 2-oxoglutarate may do the same. This is more likely to affect the details of the binding of the nicotinamide moiety than the adenosine, but clearly more structural information would now be helpful. 

Overall the targeted mutations described here altered coenzyme specificity, as predicted, in the direction of NADPH. The double mutant, in particular, behaves essentially as a dual-specific enzyme. Further mutations in this area might further encourage productive NADPH binding and, with judicious combination of mutations, it should be possible to achieve a more complete reversal of coenzyme preference. These observations serve to underline that our understanding of coenzyme specificity in dehydrogenases is as yet only partial, and there is an urgent need for more binary complex structures. 

## Figures and Tables

**Table 1:** Sequences of oligonucleotides used in this study.

Name	Sequence 5′-3′	Corresponding position in wildtype gene
A242Gf	GCGCCCCATMCTACGTTACC	921–940
A242Gr	GGTAACGTAGKATGGGGCGC	921–940
F238Sf	GCATTAGCAGGTTCTGGTAACGTAGCATGGGG	906–937
F238Sr	CCCCATGCTACGTTACCAGAACCTGCTAATGC	906–937
P262Sf	GTTACACTTTCCGGATCAGACGGATACATCTACG	974–1007
P262Sr	CGTAGATGTATCCGTCTGATCCGGAAAGTGTAAC	974–1007
D263Kf	CAGTTACACTTTCCGGACCAAAGGGATACATCTACGATCCG	973–1012
D263Kr	CGGATCGTAGATGTATCCCTTTGGTCCGGAAAGTGTAACTG	973–1012
Seq	TCCTATGGGTGGTGCCAAAG	563–582

**Table 2:** Comparison of kinetic parameters between wildtype, and F238S, P262S and F238S/P262S mutant enzymes. To determine kinetic parameters for NAD(P)H, NH_4_Cl, and oxoglutarate concentrations were kept constant at 100 mM and 20 mM, respectively, over a range of NAD(P)H concentrations (0.001–0.3 mM) under standard assay conditions. All experiments were repeated in triplicate, and the kinetic parameters and their standard errors (±SE) were calculated by the Wilkinson nonlinear regression method [10] with Enzpack version 3.0 (Biosoft Ltd., UK).

			NADH			NADPH	
	pH	*k*_cat_ (s^−1^)	*K*_*m*_ (mM)	*k*_cat_/*K*_*m*_ (s^−1^ mM)	*k*_cat_ (s^−1^)	*K*_*m*_ (mM)	*k*_cat_/*K*_*m*_ (s^−1^ mM)
Wildtype	6.0	19.9 ± 0.3	0.036 ± 0.011	553	3.59 ± 0.23	0.061 ± 0.01	58.9
A242G	6.0	11.2 ± 1.3	0.034 ± 0.010	329	1.43 ± 0.01	0.074 ± 0.01	19.3
F238S	6.0	13.6 ± 0.7	0.028 ± 0.004	485	5.00 ± 0.33	1.0 ± 0.02	5.00
P262S	6.0	14.4 ± 1.0	0.018 ± 0.005	800	14.8 ± 1.1	1.1 ± 0.16	13.5
F238S/P262S	6.0	13.9 ± 1.1	0.048 ± 0.012	289	11.2 ± 1.5	2.00 ± 0.46	5.75
D263K	6.0	14.6 ± 0.6	0.022 ± 0.001	663	20.0 ± 1.6	0.068 ± 0.010	294

Wildtype	7.0	130.2 ± 3.1	0.020 ± 0.001	6500	2.87 ± 0.11	0.090 ± 0.011	31.9
A242G	7.0	65.1 ± 1.6	0.025 ± 0.001	2604	1.87 ± 0.01	0.386 ± 0.05	4.84
F238S	7.0	139 ± 5	0.040 ± 0.003	3475	11.1 ± 0.21	0.23 ± 0.05	48.3
P262S	7.0	160 ± 8	0.047 ± 0.103	3404	62.5 ± 1.6	0.11 ± 0.01	568
F238S/P262S	7.0	105 ± 4	0.035 ± 0.010	3000	83.3 ± 3.5	0.33 ± 0.02	252
D263K	7.0	80 ± 2.5	0.023 ± 0.002	3478	26.3 ± 0.35	0.107 ± 0.02	246

Wildtype	8.0	232 ± 22	0.078 ± 0.013	2974	0.82 ± 0.11	0.53 ± 0.07	1.54
A242G	8.0	177 ± 9	0.085 ± 0.010	2082	~0.15	ND	ND
F238S	8.0	402 ± 15	0.230 ± 0.018	1747	3.61 ± 0.21	0.462 ± 0.087	7.81
P262S	8.0	490 ± 37	0.142 ± 0.016	3450	88.5 ± 1.15	0.330 ± 0.108	268
F238S/P262S	8.0	133 ± 3	0.204 ± 0.064	665	142 ± 2.2	0.56 ± 0.12	221
D263K	8.0	150 ± 11	0.091 ± 0.010	1648	37.5 ± 0.17	0.154 ± 0.06	232

**Table 3:** Summary, for each enzyme at each pH, based on data from Table 2, of the extent of discrimination against NADPH and in favour of NADH. The figure, given in the third column, is calculated as the ratio of the catalytic efficiency, *k*_cat_/*K*_*m*_, with NADH to that with NADPH. These ratios for each mutant are then in turn compared with the figure for the wildtype enzyme to obtain the figures in the fourth column, which indicate the extent to which the mutations have reversed the preference for NADH.

Enzyme	pH	NADH/NADPH preference	Shift in preference
Wildtype	6.0	9.4	—
F238S	6.0	97	0.096x
P262S	6.0	59	0.16x
F238S/P262S	6.0	50	0.19x
D263K	6.0	2.3	4.1x

Wildtype	7.0	203	—
F238S	7.0	71.9	2.2x
P262S	7.0	6.0	26.2x
F238S/P262S	7.0	11.9	13.2x
D263K	7.0	14.1	11.1x

Wildtype	8.0	1930	—
F238S	8.0	224	8.6x
P262S	8.0	12.9	150x
F238S/P262S	8.0	3.0	643x
D263K	8.0	7.1	272x
